# Effect of the G72 (DAOA) putative risk haplotype on cognitive functions in healthy subjects

**DOI:** 10.1186/1471-244X-9-60

**Published:** 2009-09-24

**Authors:** Andreas Jansen, Sören Krach, Axel Krug, Valentin Markov, Thomas Eggermann, Klaus Zerres, Markus Thimm, Markus M Nöthen, Jens Treutlein, Marcella Rietschel, Tilo Kircher

**Affiliations:** 1 Section of BrainImaging, Department of Psychiatry and Psychotherapy, Philipps-University Marburg, Germany; 2 Department of Psychiatry and Psychotherapy, RWTH Aachen University, Germany; 3 Institute of Human Genetics, RWTH Aachen University, Germany; 4 Department of Genomics, Life & Brain Center, University of Bonn, Germany; 5 Division of Genetic Epidemiology in Psychiatry, Central Institute of Mental Health, Germany; 6 Department of Psychiatry and Psychotherapy, Philipps-University Marburg, Germany

## Abstract

**Background:**

In the last years, several susceptibility genes for psychiatric disorders have been identified, among others *G72 *(also named D-amino acid oxidase activator, DAOA). Typically, the high-risk variant of a vulnerability gene is associated with decreased cognitive functions already in healthy individuals. In a recent study however, a positive effect of the high-risk variant of *G72 *on verbal working memory was reported. In the present study, we therefore examined the relationship between *G72 *genotype status and a broad range of cognitive functions in 423 healthy individuals.

**Methods:**

The *G72 *carrier status was assessed by the two single nucleotide polymorphisms (SNPs) M23 and M24. Subjects were divided into three risk groups (low, intermediate and high risk).

**Results:**

*G72 *status influenced a number of cognitive functions, such as verbal working memory, attention, and, at a trend level, spatial working memory and executive functions. Interestingly, the high-risk allele carriers scored better than one or even both other groups.

**Conclusion:**

Our data show that the putative high-risk haplotype (i.e. homozygote C/C-allele carriers in SNP M23 and homozygote T/T-allele carriers in SNP M24) is in healthy individuals not necessarily associated with worse performance in cognitive functions, but even with better performance in some domains. Further work is required to identify the mechanisms of *G72 *on brain functions.

## Background

Cognitive functions are impaired in schizophrenia [1,2] and, to a lesser extent, also in bipolar disorder [3,4] and major depression [5]. Among the different cognitive domains, verbal memory, verbal fluency and attention are typically most affected [2,3,5,6]. Especially in schizophrenia, these impairments are already present in adolescence, long before the onset of psychotic symptoms [4,6], in the prodromal state [7] and can also be found in relatives of patients [8,9], suggesting a genetic influence.

In the last years, several susceptibility genes for psychiatric disorders have been identified (for reviews, see [10-13]. Among these, *G72 *(recently named D-amino acid oxidase activator, DAOA) is one of the most frequently replicated vulnerability genes [14]. It shows a genetic overlap across the major psychoses, such as bipolar disorder, major depression and schizophrenia [15-21], questioning the long-held view of a strict nosological separation of psychiatric disorders [16,17].

To reveal potential in vivo functions of risk genes several studies have assessed genotype effects in healthy individuals. For several risk alleles, an association with subtle impairments in cognitive functions (e.g., [22]) or disadvantageous personality traits (e.g., [21,23,24]) have been found, although this does depend on the gene and the respective tagging marker.

In a recent study, we investigated the effect of *G72 *genotype on working memory using both neuropsychological tests and functional neuroimaging. Unexpectedly, the putative high-risk haplotype (i.e. homozygote C/C-allele carriers in the single nucleotide polymorphism (SNP) M23 and homozygote T/T-allele carriers in SNP M24) was associated with significant *better *performance in verbal working memory. These behavioural differences were accompanied by a stronger deactivation in the right parahippocampus during a working memory 2-back task [25]. Thus, the high risk variant of *G72 *has a beneficial influence on verbal working memory in healthy subjects, although it is known to increase the risk for schizophrenia and affective disorders, diseases that are associated with impairments in this domain [2,3,5,6].

In the present study we further investigated how genetic alterations in *G72 *influence cognitive functions in healthy individuals. We examined whether the positive influence of the high risk *G72 *variant is restricted to verbal working memory or whether this genotype also beneficially influences other cognitive domains.

## Methods

### Subjects

The subjects were recruited through postings at the University of Aachen, advertisements in local newspapers and an e-mail sent to all students of the University of Aachen. 423 subjects (214 men, 209 women) were included in the present study. Inclusion criteria were age (18-55 years), right-handedness (as assessed by the Edinburgh Laterality Scale, [26]), no psychiatric disorders according to ICD-10 and Western- or Middle European descent. The subjects' characteristics are given in Table 1a.

**Table 1:** G72 risk status

***G72 *risk status**	**low**	**intermediate**	**high**	**F**	**p**
**a: Subjects' characteristics**					
number of subjects	88	231	104		
Sex ratio (men/women)	43/45	115/116	56/48	χ^2 ^= 0.606	.739
Age (years)	25.0 (6.7)	24.9 (6.3)	23.8 (3.7)	1.387	.251
Education (years)	15.5 (2.6)	15.5 (2.8)	15.5 (2.2)	.005	.995

**b: Cognitive variables**					
Spatial span	19.09 (2.88)	18.82 (3.12)	19.62 (2.60)	2.585	.077
Letter-number-span test	16.36 (2.51)	16.28 (2.55)	17.32 (2.26)	6.636	.001 *
Trail Making Test	58.29 (15.01)	62.85 (19.67)	57.31 (15.42)	4.357	.013
Semantic verbal fluency	30.75 (8.88)	30.91 (9.21)	32.81 (9.63)	1.734	.178
Lexical verbal fluency	17.38 (4.25)	16.54 (4.47)	16.89 (5.08)	1.096	.335
d2-test	201.3 (35.7)	188.0 (33.5)	198.8 (33.8)	6.545	.002 *

Subjects are divided into three groups (low, intermediate and high risk) according to their G72 status (based on two SNPs, M23 and M24). (a) Subjects' characteristics: sex, age and education. There were no significant group differences in sex ratio, age or education (p > .1). (b) Cognitive results of a neuropsychological test battery testing working memory, executive functions, verbal fluency and attention. Due to Bonferroni corrections for multiple testing, a significance threshold of p = 0.008 was set as significance criterion. Standard deviations are given in parentheses. Significant results are marked with '*'.

After a complete description of the procedure subjects provided written informed consent to participate in the study. The protocol was approved by the local ethics committee according to the declaration of Helsinki. After participants provided consent, the cognitive tests were administered and blood was taken from a vein of each subject's arm.

### Genetic Analysis

Subjects were genotyped as part of a sample described in Rietschel et al. [21] for two *G72 *SNPs (M23 = rs3918342 [C/T] and M24 = rs1421292 [T/A]) using the MassARRAY^® ^system (Sequenom Inc., San Diego, Ca). For quality comparison purposes, we genotyped a subset of the sample in duplicate in order to estimate the replicate error rate. Two out of 96 DNA samples were randomly chosen for this purpose. For the SNPs genotyped, all genotypes between duplicates were consistent (0% replicate error rate). We also always include routinely positive and negative controls in our genotyping experiments. By a standard 1 df chi-square test, there were no significant deviations from Hardy-Weinberg equilibrium for the genotype distributions of the studied sample.

The association between *G72 *genotype status and psychiatric disorders was obtained for different SNPs [14,27-29]. We chose the markers M23 and M24, because the M23-M24 haplotypes C-T and T-A have recently been associated with schizophrenia, bipolar disorder, and major depression [21]. Depending on the M23 and M24 markers, the subjects were divided in three groups: low risk, intermediate risk and high risk. Subjects who had a homozygote T-allele on M23 and a homozygote A-allele on M24 were classified as "low risk". Subjects who had a homozygote C-allele on M23 and a homozygote T-allele on M24 were classified as "high risk". All other subjects belonged to the "intermediate risk" group.

In a post-hoc analysis, we additionally analysed all data separately for group classifications depending solely on the M23 and M24 status, respectively. The principal results did not change (see appendix and tables 2 and 3). This is not surprising, since both markers are highly correlated (r = 0.94).

**Table 2:** Risk status calculated by M23

***G72 *risk status (M23)**	**low**	**intermediate**	**high**	**F**	**p**
**a: Subjects' characteristics**					
number of subjects	95	221	107		
Sex ratio (men/women)	49/46	108/113	57/50	0.607	.738
Age (years)	24.9 (6.5)	25.0 (6.4)	23.8 (3.6)	1.592	.205
Education (years)	15.6 (2.6)	15.5 (2.8)	15.5 (2.2)	0.068	.935

**b: Cognitive variables**					
Spatial span	19.19 (2.85)	18.80 (3.14)	19.53 (2.65)	2.304	.101
Letter-number-span test	16.41 (2.49)	16.25 (2.58)	17.28 (2.25)	6.496	**.002**
Trail Making Test	58.41 (14.62)	62.81 (19.95)	57.73 (15.39)	3.804	0.23
Semantic verbal fluency	30.76 (8.72)	31.00 (9.31)	32.57 (9.61)	1.277	.280
Lexical verbal fluency	17.25 (4.27)	16.57 (4.48)	16.87 (5.05)	0.753	.472
d2-test	200.9 (35.0)	187.6 (33.4)	198.8 (34.3)	6.832	**.001**

**Table 3:** Risk status calculated by M24:

***G72 *risk status (M24)**	**low**	**intermediate**	**high**	**F**	**p**
**a: Subjects' characteristics**					
number of subjects	88	220	115		
Sex ratio (men/women)	43/45	109/111	62/53	0.709	.702
Age (years)	25.0 (6.7)	24.8 (6.2)	24.1 (4.4)	0.718	.489
Education (years)	15.5 (2.6)	15.5 (2.9)	15.6 (2.2)	0.010	.990

**b: Cognitive variables**					
Spatial span	19.09 (2.88)	18.84 (3.15)	19.50 (2.62)	1.900	.151
Letter-number-span test	16.36 (2.52)	16.23 (2.55)	17.31 (2.28)	7.600	**.001**
Trail Making Test	58.29 (15.00)	62.84 (19.50)	57.85 (16.34)	3.845	.022
Semantic verbal fluency	30.75 (8.88)	30.86 (9.22)	32.72 (9.56)	1.754	.174
Lexical verbal fluency	17.38 (4.25)	16.47 (4.53)	16.98 (4.90)	1.348	.261
d2-test	201.3 (35.7)	187.7 (33.7)	198.4 (33.4)	6.663	**.001**

### Neuropsychological test battery

We assessed working memory, executive functions, verbal fluency and attention. Working memory was measured with the spatial span of the Wechsler Memory Scale (spatial working memory, [30]) and with the letter-number-span test (verbal working memory, [31]). Executive functions were assessed with the Trail Making Test (TMT-B, [32]). Verbal fluency was measured with semantic and lexical word generation [33]. Attention was assessed with the d2-test [34].

Behavioural data were analyzed using a univariate ANOVA design with *G72 *status (low, intermediate and high risk) as factor between subjects and outcome of the cognitive assessments as dependent variables. Bonferroni correction was applied to correct for multiple statistical testing (six tests, p = 0.008). In a post-hoc analysis, we additionally included age as covariate (since age is known to be significantly correlated with most of the dependent variables). The principal results however did not change.

## Results

The ANOVA showed a significant (p < 0.008) main effect of *G72 *status on verbal working memory (p = 0.001, the high risk group performed better than both other groups) and attention (p = 0.002, the intermediate risk group performed worse than both other groups). Furthermore, there was a trend (p < 0.1) effect of *G72 *status on spatial working memory (p = 0.077, the high risk group performed better than both other groups) and executive function (p = 0.013, the intermediate risk group performed worse than both other groups) (Table 1b).

## Discussion

In the present study we investigated the effect of *G72 *genotype on cognitive functions in a large sample of healthy individuals. Our results show that *G72 *status influences the performance in a number of cognitive domains (significant differences in verbal working memory and attention, differences on a trend level in spatial working memory and executive functions). Most importantly, the high-risk allele carriers scored significantly better than one or even both other low-risk groups. Thus, healthy individuals with a *G72 *haplotype that is known to increase the risk for the major psychoses perform better in some cognitive domains than subjects with a low risk status, although these cognitive domains are negatively affected by the psychiatric disorders that are associated with this allele variant.

Only few studies assessed so far the effect of genetic variation in *G72 *on cognitive functions. Goldberg et al. investigated the relationship between several SNPs in the *G72 *region and select cognitive measures in attention, working memory, and episodic memory in a cohort of over 600 subjects, including patients with schizophrenia, their unaffected siblings, and healthy controls. The authors showed for the markers M23 and M24 a significant genotype by diagnosis interaction with a number of cognitive measures (working memory, attention, verbal learning). The low risk homozygote A/A genotype group scored better than the high risk T/T homozygote group, most notably in the schizophrenia group [35]. Although the authors also report a main effect of genotype at least for marker M24, this effect seems to be mainly driven by the patient sample. Opgen-Rhein and colleagues investigated the influence of *G72 *variation on cognitive performance in a large sample of both patients schizophrenia (n = 178) and healthy controls (n = 144) [36]. They showed that a certain *G72 *haplotype located upstream of the presumed gene borders of *G72 *has an impact on semantic fluency. Interestingly, carriers of the risk haplotype showed *better *semantic fluency than non-carriers, both in the patients and the control population. Donohue and colleagues report that a functional polymorphism within *G72 *(rs 2391191, M15) was associated with poorer verbal memory performance among patients with schizophrenia [37]. Taken together, these studies show that functional polymorphisms in the *G72 *gene region have an impact on cognitive functions. This impact seems to be most notable in psychiatric samples. Our study further extends these previous findings and show that the SNPs in the *G72 *gene complex have also an impact on cognitive functions in healthy controls.

Our results suggest that, at least for markers M23 and M24, the high-risk genotype of *G72 *has no negative effect on cognitive functions in healthy individuals *per se*, but even a positive effect in some cognitive domains (such as verbal working memory and attention). This finding is at first glance counterintuitive, but might be explained by a number of reasons. First, the M23-M24 risk haplotype might influence cognitive functions independent of its role as a risk factor for psychiatric disorders. A similar explanation has been proposed by Opgen-Rhine and colleagues who also report that a risk haplotype in the *G72 *region is associated with better performance in semantic processing both in patients with schizophrenia and control subjects [36]. Second, from a standpoint of evolutionary theory, it might be argued that the risk variant of *G72 *is maintained in the population since it has a beneficial influence on cognitive functions which has a positive effect for evolutionary selection [36]. At last, it cannot be fully excluded that at least some of the results represent false positive findings. It is for instance in particular difficult to understand why the intermediate risk group has a significant worse performance in the d2-test in comparison to both the high- and the low-risk group. A limitation of our study is that we cannot give a stringent neurobiological explanation for these findings. However, all results are based on a large cohort (n = 423), were obtained by stringent statistical analyses and survived Bonferroni corrected thresholds, reducing the likelihood of this interpretation.

The functional mechanisms of *G72 *are still not fully understood. Chumakov and colleagues showed that the *G72 *protein (which is only known in higher primates) activates a second protein, D-amino acid oxidase (DAAO), that is involved in the mechanisms of D-serine [38]. D-serine is an agonist at the glycine modulation site of the N-methyl-D-aspartate (NMDA) receptor [39]. Thus, *G72 *might work as an indirect modulator of NMDA neurotransmission, which has been implicated in various cognitive domains. Lower serum level of D-serine has been shown, for instance, in patients with schizophrenia. Furthermore, the administration of D-serine (as add-on medication) has been shown to reduce some of the symptoms in schizophrenia [40]. This provides a potential link between *G72 *and the glutamate hypofunction hypothesis of schizophrenia [41]. Another study however failed to confirm the interaction between *G72 *and DAAO [42]. Rather, LG72, a splicing isoform of the *G72 *gene, encodes for a mitrochondrial protein. It was shown that an overexpression of *G72 *led to mitrochondrial fragmentation. The authors proposed that an unknown function of the *G72 *in modulating mitochondrial morphology might be responsible for the risk-conferring property of the gene.

Several fMRI studies suggest a modulatory role of *G72 *on brain activity in the medial temporal lobe (MTL), in particular the hippocampus and parahippocampus [25,35,43]. Goldberg and colleagues showed that healthy control subjects carrying the homozygous high-risk T/T allele at SNP M24 had decreased brain activity of the right hippocampus and left parahippocampus during an episodic memory encoding task [35]. Hall et al. investigated subjects with a high familial risk for schizophrenia and report brain activation differences related to the *G72 *genotype (as assessed by SNPs M23 and M24) in the left hippocampus and parahippocampus during a verbal sentence completion task [43]. Jansen and colleagues showed that in healthy control subjects the *G72 *genotype (determined by SNPs M23 and M24) is correlated with brain activity of the right parahippocampus during a working memory task [25].

## Conclusion

Taken together, these findings can be summarized as follows:

1. *G72 *is a vulnerability gene for several psychiatric disorders, including schizophrenia, bipolar disorder, major depression, and panic disorder [14]. However, about 25% of the general population, as suggested by the present study, carry the high-risk-variant, making a direct negative effect of the "high-risk" haplotype of *G72 *unlikely.

2. The high-risk variant increases the risk for cognitive impairments in patients with schizophrenia, that is, when the disorder is already in an acute state [35]. However, the high-risk haplotype does not negatively affect cognitive abilities *per se*, but has a beneficial influence on some cognitive functions in healthy individuals (as shown in the present study). This might be one reason, why the allele has not been selected out during evolution.

3. Functional imaging studies suggest a modulatory influence of *G72 *on brain activity in the MTL (hippocampus, parahippocampus) [25,35,43]. These structures are involved in the pathogenesis of affective disorders and particularly schizophrenia [44,45].

The mechanism of *G72 *might therefore be explained by the following hypothesis:

*G72 *has a modulatory influence on brain activity in the MTL. The high-risk variant has overall a positive effect on cognitive abilities, but also increases the risk, in combination with other (unknown) genetic and epigenetic factors, to increase the risk for psychiatric disorders via its modulatory influence on the MTL structures.

## Competing interests

The authors declare that they have no competing interests.

## Authors' contributions

AJ performed the statistical analysis, was involved in the interpretation of data, made substantial contributions to conception and design and drafted the manuscript. SK was involved in the statistical analysis and the interpretation of data and helped to draft the manuscript. AK was involved in the acquisition of data, made substantial contributions to conception and design and was involved in drafting the manuscript. VM was involved in the acquisition of data and was involved in drafting the manuscript. MT was involved in the statistical analysis and the interpretation of data and helped to draft the manuscript. TE was involved in the genetic analyses and was involved in drafting the manuscript. KZ was involved in the genetic analyses and was involved in drafting the manuscript. MN made substantial contributions to conception and design, was involved in the genetic analyses. JT was involved in the genetic analyses and was involved in drafting the manuscript. MR made substantial contributions to conception and design and was involved in the genetic analyses. TK conceived of the study, and participated in its design and coordination and helped to draft the manuscript. All authors read and approved the final manuscript.

## Appendix

In a post-hoc analysis, we additionally analysed all data separately for group classifications depending solely on the M23 and M24 status, respectively. The principal results did not change. This is not surprising, since both markers are highly correlated (r = 0.94). In this appendix, we additionally present the results of these analyses. See tables 2 and 3.

## Pre-publication history

The pre-publication history for this paper can be accessed here:


